# Redefining the Inclusion Criteria for Successful Steindler Flexorplasty Based on the Outcomes of a Case Series in Eight Patients

**DOI:** 10.1055/s-0043-1767672

**Published:** 2023-10-11

**Authors:** Alexander A. Gatskiy, Ihor B. Tretyak, Jörg Bahm, Vitaliy I. Tsymbaliuk, Yaroslav V. Tsymbaliuk

**Affiliations:** 1 Restorative Neurosurgery Department, Romodanov Neurosurgery Institute, Kyiv, Ukraine; 2 Klinik für Plastische Chirurgie, Hand- und Verbrennungschirurgie, Sektion Plexuschirurgie in der Uniklinik RWTH Aachen, Aachen, Deutschland; 3 National Academy of Medical Sciences of Ukraine, Kyiv, Ukraine; 4 Group of Chronic Pain Treatment, Romodanov Neurosurgery Institute, Kyiv, Ukraine

**Keywords:** brachial plexus injury, nerve transfer, Steindler flexorplasty

## Abstract

**Background (rationale)** Steindler flexorplasty (SF) is aimed at restoring independent elbow flexion in the late stages of dysfunction of the primary elbow flexors. Selection criteria for successful SF have been defined.

**Objectives** The purpose of this study was to redefine the inclusion criteria for successful SF based on functional outcomes.

**Methods** Eight patients received SF after an average of 50.8 months after injury or dysfunction. Three patients (37.5%) met all five Al-Qattan inclusion criteria (AQIC), and another five patients (62.5%) met four or less AQIC. Patients were followed up for at least 9 months, and the maximum range of active elbow flexion (REF) was measured. Functional results of SF were assessed using the Al-Qattan scale (in accordance with Al-Qattan's scale).

**Results** The mean maximum REF was 100 degrees (70 to 140 degrees). Five patients reached REF greater than 100 degrees. One patient had a poor outcome, two patients (25%) had a fair outcome, three patients (37.5%) had a good outcome, and two patients (25%) had an excellent outcome of SF on the Al-Qattan scale. The impact of each AQIC on functional outcome has been critically reviewed from a biomechanical point of view.

**Conclusions** The sufficient number of inclusion criteria required for successful SF can be reduced from five (according to AQIC) to two; Normal or near-normal function (M4 or greater on the MRC scale) of the muscles of the flexor-pronator mass should be considered an obligatory inclusion criterion, while primary wrist extensors may be considered an optional inclusion criterion.

## Introduction

It has been established that the reconstruction of the independent elbow flexion (EF) has the highest priority among all functions of the upper extremity. 1 Conditions under which the primary elbow flexors lose their function are as follows: myelopathy, radiculopathy, plexopathy, and neuropathy of various etiologies. 2 Regardless of the etiology of dysfunction, there are only two principal ways to restore independent EF—nerve 3 4 or tendon and muscle 5 6 surgery. Since the first one is strongly adhered to the time factor, 7 the second one, which is aimed to restore independent EF, is not affected by the latter. 5 6 Since functional free muscle transfers 8 remain the prerogative of highly specialized microsurgical centers with a long history of successful utilization of the technique with low morbidity rate of the transferred myocutaneous flap, 9 approaches to the reconstruction of an effective EF remain dependent on the two abovementioned “classical” techniques, 3 4 5 6 available to most surgeons.

Nowadays, transfers of a single muscle 5 6 or entire muscle complexes 5 6 are majorly indicated in an event of completely or partially failed reinnervation or in the later stages of the disease when surgically induced reinnervation obviously fails. 5 6

Unipolar or bipolar pectoralis major, 5 6 latissimus dorsi, 5 6 triceps, 5 6 and the flexor-pronator mass of the forearm, known as Steindler flexorplasty (SF), 10 muscle transfers are considered to be the most effective techniques aimed at restoring independent EF at later stages.

One of the earliest procedures 10 and still preferred by many surgeons 11 is SF and its multiple modifications. 12 13 14 15 16 As the technical details of the procedure itself are well described in numerous publications from around the world 12 13 14 15 16 in a simplified manner, the procedure involves changing the cranial attachment of the flexor-pronator mass of the forearm to form humeral medial epicondyle to a more proximal point on the anterior surface of the humeral shaft. 11 Contraction of the flexor-pronator mass of the forearm should produce EF with its cranial attachment proximalized.

It is well known that under normal conditions, several of the most powerful muscles of the flexor-pronator mass of the forearm (namely the flexor carpi radialis and flexor carpi ulnaris) act across both adjacent joints—the wrist (to a greater extent) and elbow (to a lesser extent). 17 Ideally, reattachment of the primary wrist flexors during SF should produce an increase in the range of active elbow flexion (REF) during contraction. In real-world conditions, shortening of the bellies of reattached muscles is accompanied by wrist flexion (distal attachment point) and is clinically manifested by a condition known as “active insufficiency” 17 during EF. The wrist extensor muscles serve as a “fuse” in case of “active insufficiency” of the transferred flexor-pronator mass of the forearm, reversing this state into a “passive insufficiency” when they are stretched. 17 Passive insufficiency of the primary wrist flexors during wrist extension causes the elbow to flex, while the transferred flexor-pronator mass contracts and shortens. Preserved functioning of the wrist extensors serves as the main selection criterion for a successful SF. 18

Al-Qattan defined 19 other valuable selection criteria for successful SF. These include a stable shoulder, a strong hand grip (at least M4 on the MRC scale 20 ), strong wrist and elbow extension (at least M4 on the MRC scale), and the presence of the “Steindler effect'' 19 . According to Al-Qattan 19 “Steindler effect” is achieved by pronating the forearm and flexing the wrist, and then swinging the arm at the elbow to overcome gravity while attempting EF. The overall success of the procedure is influenced not only by preoperative criteria. 19 Side effects associated with SF, both static and dynamic in origin, have the potential to change the outcome. 19 Static side effects include EF contracture, pronation contracture, wrist flexion contracture. 19 Dynamic side effects include clenched fist syndrome and wrist hyperflexion. 19

The purpose of this study was to redefine the inclusion criteria based on functional outcomes in the case series and describe the pathological locomotor phenomena (PLP) associated with SF and their impact on functional outcomes.

## Methods

### Study Design and Patient Population Characteristics

A retrospective descriptive study was conducted based on a case series of eight patients from 2015 to 2021. The study included eight patients (three women and five men) aged 20 to 73 years (mean age 40.5; Table 1 ). Five patients had dysfunction (complete palsy, M0 on the MRC scale) of the primary elbow flexors (biceps brachii and brachialis) associated with brachial plexus injury as a result of a motorcycle accident, one patient additionally had a severe craniocerebral injury ( Table 1 ). The anatomy of the brachial plexus injury at the time of admittance was as follows: two cases of C5 to C6 and three cases of C5 to C6 to C7 injury ( Table 1 ). In two patients, the function of the primary elbow flexors was lost due to iatrogenic issues—the removal of a benign extramedullary spinal cord tumor and a brachial plexus tumor ( Table 1 ). One patient had a history of cervical herniated disc surgery followed by anterior cervical interbody fusion (ACIF) and irreversible C6 radiculopathy ( Table 1 ).

**Table 1:** Patient population characteristics

#	Gender/Age	Type of injury	Primary surgery	Neurologic deficit at admittance	Time to PNS	Primary nerve surgery (PNS)	Time to SNS	Secondary nerve surgery (SNS)
1	F/46	BP tumor	Tumor removal (2011)	C5 − C6	**7** **mo**	C5 + C6-C6 grafting (2012)	−	−
2	M/31	BPI	−	C5 − C6 − C7	**3 mo**	BP neurolysis (2016)	**14 mo**	RN-Ax; Acc-SS (2018)
3	F/20	BPI/CCT	CCT (2017)	C5 − C6 − C7	**12 mo**	Acc-SS; Acc-Ax (2018)	−	−
4	M/40	BPI	−	C5 − C6	**5 mo**	Pect(M)-Msc; Acc-SS; ThD-ThL(2017)	−	−
5	M/73	Radiculopathy	ACIF (2018)	C6	−	−	−	−
6	F/47	Cervical tumor	Tumor removal (2015)	C5 − C6 − C7	**16 mo**	RN-Ax; Acc-SS (2017)	−	−
7	M/45	BPI	−	C5 − C6	**3 mo**	BP neurolysis (1999)	−	−
8	M/21	BPI	−	C5 − C6 − C7	**11 mo**	PhN-Msc (2021)	−	−

Note: Acc, spinal accessory nerve; ACIF, herniated disc surgery, anterior cervical interbody fusion; Ax, axillary nerve; BP, brachial plexus; BPI, brachial plexus injury; CCT, craniocerebral trauma; F, female; M, male; Msc, musculocutaneous nerve; PhN, phrenic nerve; RN, radial nerve; SS, suprascapular nerve; ThD, thoracodorsal nerve; ThL, long thoracic nerve

Seven patients underwent primary nerve surgery (PNS) within 3 to 16 months (average 8 months), and only one patient did not undergo nerve surgery after ACIF ( Table 1 ). Four patients received nerve transfer procedures as PNS within 5 to 16 months after injury ( Table 1 )—late reconstruction, while two patients received nerve transfers to reinnervate the primary elbow flexors (5 and 11 months after injury) and another two received nerve transfers to restore only the deltoid muscle and external rotators of the shoulder (12 and 16 months after injury) ( Table 1 ). Another three patients received PNS, which consisted of neurolysis (two cases) of the brachial plexus and grafting of the C5 to C6 trunk in the supraclavicular region (3, 3, and 7 months after injury, respectively; Table 1 ).

One patient with brachial plexus injury received secondary nerve surgery (SNS) due to lack of recovery of EF and shoulder abduction/external rotation at 11 months after PNS and 14 months after injury ( Table 1 ). All nerve transfer procedures were conducted in accordance with all requirements for donor and acceptor nerves, 21 except for the timing 7 of the procedures. The performed procedures are schematically presented in Table 1 .

Overall, the neurological status of the injured upper extremity (outcomes of PNS and SNS) was assessed at the time of inclusion and after an average of 50.8 months (12 to 240 months) after injury or dysfunction ( Table 2 ).

**Table 2:** Functional assessment of the muscles of the corresponding segments (units) of the upper extremity at the time of inclusion in the study

#	Year of inclusion	Time to inclusion	SH Unit		E Unit			HW Unit	
			SFF	ABD	EF	BR	TBM	WE	WF/FF
1	2015	3.5 y	90 degrees	90 degrees	M0	M4	M5	M5	M5
2	2018	2 y	30 degrees	0 degrees	M0	−	M4 a	M3 a	M5
3	2018	14 mo	0 degrees	0 degrees	M0	−	M2	M2	M4
4	2018	1.5 y	0 degrees	45 degrees	M0	M4	M4	M4	M5
5	2019	1 y	180 degrees	120 degrees	M0	M5	M5	M5	M5
6	2019	3.5 y	30 degrees	20 degrees	M0	−	M3	M2	M4
7	2019	20 y	0 degrees	0 degrees	M0	M4	M5	M5	M5
8	2021	13 mo	45 degrees	0 degrees	M0	−	M3	M4	M4

Abbreviations: ABD, shoulder abduction; BR, brachioradialis muscle; E, elbow unit; EF, elbow flexion (provided by primary elbow flexors); FF, finger flexion; HW, hand and wrist unit; M, power of the elbow flexion according to the UK MRC; SFF, shoulder forward flexion; SH, shoulder unit; TBM, triceps brachii muscle; WE, wrist extension; WF, wrist flexion.

a Proximal-distal sequential (non-antagonistic) cocontraction associated with aberrant regeneration of a brachial plexus injury.

The two main study inclusion criteria (along with the absence of contraindications to surgery associated with the somatic status of the patient and the absence of soft tissue infection of the injured upper extremity) were as follows: (1) no function of the primary elbow flexors (biceps brachii and brachialis), which was electromyography confirmed; (2) clinically strong functions of the muscles of the flexor-pronator mass (namely, the pronator teres, flexor carpi radialis, and ulnaris) of the anterior surface of the forearm (M4 and greater on the MRC scale) and both superficial and deep finger flexors (M4 and greater on the MRC scale) of the injured upper extremity ( Table 3 ).

**Table 3:** Congruence of neurological functions of the injured upper extremity with different inclusion criteria among patients selected for Steindler flexorplasty in the study

	Study inclusion criteria			Al-Qattan inclusion criteria a									
#				Stable Shoulder			Strong EE		SEF	Strong hand grip		Strong WE	
		WF/FF	EF		SFF	ABD		MTB			WF/FF		ECRB/L
**1**	+	M5	M0	**+**	90 degrees	90 degrees	+	M5	+	+	M5	+	M5
**2**	+	M5	M0	−	30 degrees	0 degrees	+	M4	−	+	M5	−	M3
**3**	+	M4	M0	−	0 degrees	0 degrees	−	M2	−	+	M4	−	M2
**4**	+	M5	M0	+	0 degrees	45 degrees	+	M4	+	+	M5	+	M4
**5**	+	M5	M0	+	180 degrees	120 degrees	+	M5	+	+	M5	+	M5
**6**	+	M4	M0	+	30 degrees	20 degrees	−	M3	−	+	M4	−	M2
**7**	+	M5	M0	−	0 degrees	0 degrees	+	M5	+	+	M5	+	M5
**8**	+	M4	M0	+	45 degrees	0 degrees	−	M3	−	+	M4	+	M4

Abbreviations: ABD, shoulder abduction; ECRB/L, extensor carpi radialis brevis/longus; EE, elbow extension; EF, elbow flexion (provided by primary elbow flexors); FF, finger flexion; HW, hand and wrist unit; M, power of the elbow flexion according to the UK MRC; SEF, Steindler effect; SFF, forward flexion of the shoulder; TBM, triceps brachii muscle; WE, wrist extension; WF, wrist flexion.

a According to Al-Qattan. 19

In addition, segmental functions of the shoulder, elbow, hand, and wrist unit 17 were assessed in accordance with the preserved/recovered neurological functions (muscles) of the injured upper extremity by clinical neurological examination/electromyography and in accordance with the inclusion criteria described by Al-Qattan. 19

At the time of inclusion, the neurological conditions of the injured upper extremity met all five Al-Qattan inclusion criteria (AQIC) n three patients (37.5%). Another five patients (62.5%) met four or less AQIC: one patient met four, one patient met three, two patients met two, and one patient met one AQIC ( Table 3 ).

### Surgical Technique

**Fig. 1:**
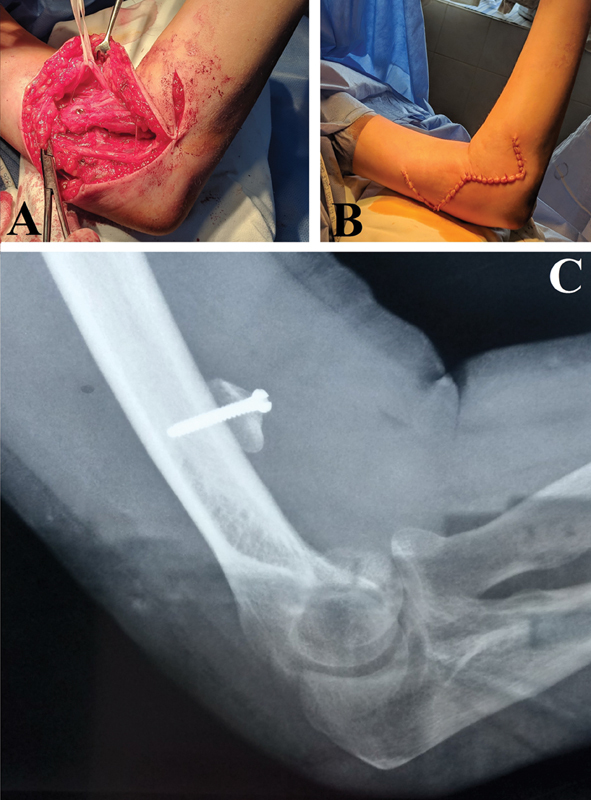
. Intraoperative and X-ray modifications of Steindler flexorplasty by Loeffler and Lewis 6 applied in the study. ( **A** ) A round-shaped bone mass of the medial epicondyle of the humerus with an attached flexor-pronator mass, transferred to the anterior aspect of the humeral shaft. ( **B** ) W-shaped skin incision after wound closure, ( **C** ) A single-unit unicortical screw fixation of the osteotomized round-shaped medial epicondyle approximately 6 cm in relation to the coronoid fossa of the humerus.

The utilized surgical technique was in a step wise accordance with that described by Loeffler and Lewis, 6 with only three main differences: (1) a W-shaped skin incision was applied ( Fig. 1, B ); (2) a round-shaped bone mass derived after osteotomy of the medial epicondyle of the humerus was at least 5 mm high and at least 1.2 cm in its diameter ( Fig. 1, A ); (3) single-screw fixation (without any additional anchors) of the osteotomized bone mass of the medial epicondyle to the anterior surface of the humeral shaft using unicortical and biocortical end screws ( Fig. 1, C ).

### Follow-up And Evaluation of Results

Postoperatively, the elbow was immobilized with plaster at the joint flexion of 90 degrees for 5 to 7 weeks. The removal of the splint was preceded by an X-ray examination without radiographic evidence of screw or bone mass dislocation. Physiotherapy exercises started immediately in the outpatient department.

The patient was followed up for at least 9 months. During the examination, REF was measured—the maximum REF was taken as the angular deviation from the position of the maximum possible elbow extension to the position of the maximum possible EF. The absence of a further increase in the REF at two repeated examinations over a period of at least 3 months was considered the final outcome. The time from initial injury to maximum REF was assessed. The functional results of SF were assessed according to Al-Qattan's scale. ( Table 4 ).

**Table 4:** Assessment of results following Steindler flexorplasty according to Al-Qattan 19

Result	Criteria
Excellent	There is greater than 120 degrees active elbow flexion against resistance and less than 20 degrees of elbow extension loss.
Good	There is 100–120 degrees of active elbow flexion against resistance and/or the elbow flexion contracture is between 20 and 40 degrees.
Fair	Active elbow flexion against resistance is less than 100 degrees and/or elbow flexion contracture is greater than 40 degrees.
Poor	No active elbow flexion or unimproved elbow flexion (failed procedure). a

a All combined in this study: elbow flexion only against gravity and less than 90 degrees and elbow flexion contracture greater than 40 degrees.

The presence of irreversible contractures (abnormal postures 19 ) was documented at the time of the final outcome assessment. The presence of only EF contracture affected the scoring during the final outcome assessment according to Al-Qattan's scale. 19 Along with documenting occurrence of static abnormal postures, dynamic PLP in the hand and wrist unit were documented. These PLP were described in analogy to the preoperative Steindler effect. 19 At the time of the final outcome assessment, the presence of wrist flexion during active EF was regarded as a direct Steindler effect and wrist extension during active EF as a reverse Steindler effect. The influence of both types of PLP on the opening and closing of the hand in the event of maximum EF was determined. The inability to open the hand (fist clench 19 ) with an elbow flexed in the presence of either direct or reverse PLP was considered parasitic, having a pure negative effect on the functionality of the hand and wrist unit of the injured upper extremity. Changes in preoperative or postoperative transverse volar grip strength were not documented in this series. The presence of either PLP was not included in the scoring process during the final evaluation of results.

## Results

The mean time from injury to maximum REF after SF was approximately 5 years (15 months to 21 years; Table 5 ).

**Table 5:** Abnormal postures (static) in segments of the upper extremity and pathological locomotor phenomena (dynamic abnormal posture) in the hand and wrist unit (HWU) after Steindler flexorplasty

#	EFC/EE	PC	FC	WFC	HWU PLP	Time to maximal EF (from FLXRP/injury)	EF	MRC	Result a
1	N/180 degrees		Y		Reverse SEF	∼2 y (∼5.5 y)	100 degrees	M4	Good
2	20 degrees /160 degrees	Y	Y	Y	Direct SEF	∼1 y (∼3 y)	80 degrees	M4	Fair
3	30 degrees /150 degrees	Y		Y	Direct SEF	6 mo (∼2 y)	90 degrees	M4	Fair
4	N/180 degrees		Y		Reverse SEF	6 mo (∼2 y)	100 degrees	M4	Good
5	N/180 degrees				F and W neutral	3 mo (15 mo)	140 degrees	M4	Excellent
6	50 degrees /130 degrees	Y	Y	Y	Direct SEF	∼1 y (∼4.5 y)	70 degrees	M3	Poor
7	10 degrees /170 degrees		Y		Reverse SEF	9 mo (∼21 y)	120 degrees	M4	Excellent
8	40 degrees /140 degrees	Y	Y		W neutral	9 mo (21 mo)	100 degrees	M4	Good

Abbreviations: EE, elbow extension (both active and passive); EF, elbow flexion after Steindler flexorplasty; EFC, elbow flexion contracture; F, fingers; FC, fist clench; HWU, hand and wrist unit; N, no; PC, pronation contracture; PLP, pathological locomotor phenomena; SEF, Steindler effect; W, wrist; WFC, wrist flexion contracture; Y, yes.

a According to Al-Qattan. 19

The mean time from SF to maximum REF was approximately 10 months (3 to 24 months; Table 5 ). Seven out of eight patients achieved maximum REF within the first 12 months ( Table 5 ), five patients achieved maximum REF in less than 9 months and three patients achieved maximum REF in less than 6 months after SF ( Table 5 ).

The mean REF, regardless of the time from injury or SF to the maximum REF, was 100 degrees (70 to 140 degrees; Table 5 ), with five patients able to produce the EF more than 100 degrees, which was considered good or excellent outcome.

Seven out of eight patients, regardless of the time from injury or SF, to the maximum REF, were able to perform against resistance (M4 on the MRC scale; Table 5 ), which was considered as good or excellent outcome.

Only one patient didn't develop any static abnormal postures after SF ( Table 5 ). Another seven patients had various static abnormal postures: elbow extension contracture in five cases (range 10 to 50 degrees and mean = 30 degrees), pronation contracture in four cases, fist clench in six cases, and wrist flexion contracture in three cases ( Table 5 ).

Overall, only one patient (12.5%) had a poor outcome due to a combination of all three outcome assessment criteria according to Al-Qattan's scale ( Table 4 ): severe elbow extension contracture (approximately 50 degrees), maximum REF (less than 70 degrees) with no action against resistance (M3 on the MRC scale) ( Table 5 ). Two patients (25%) had a fair outcome due to a combination of two outcome assessment criteria according to Al-Qattan's scale ( Table 4 ): elbow extension contracture (20 and 30 degrees, respectively) and a maximum REF of 80 and 90 degrees against resistance (M4 on the MRC scale; Table 5 ). Three patients (37.5%) had a good outcome, primarily due to a restricted maximum REF against resistance (M4 on the MRC scale) of approximately 100 degrees in all cases ( Table 5 ), and two of them had no accompanying elbow extension contracture ( Table 5 ).

Two patients (25%) had an excellent outcome and were able to produce maximum REF against resistance (M4 on the MRC scale) of approximately 120 and 140 degrees ( Table 5 ), and only one of them had a minor accompanying elbow extension contracture of 10 degrees ( Table 5 ).

Only one patient had no pathologic locomotor phenomena (dynamic abnormal posture) in the hand and wrist unit after SF ( Table 5 )—the patient was able to maintain a neutral wrist and finger position during active EF. Another patient was able to maintain a neutral wrist position during active EF, but only with his fist clenched ( Table 5 ). The ability to maintain a neutral wrist position occurred in a subgroup of patients with good and excellent outcomes.

In a subgroup of three patients with PLP, considered in this study as a direct “Steindler effect”—EF accompanied by excessive wrist flexion—the overall outcome was predominantly below average (fair in two cases and poor in one case; Table 5 ). In a subgroup of three patients with PLP, considered in this study as a reverse “Steindler effect”—EF accompanied by excessive wrist extension—the overall outcome was predominantly good to excellent (good in two cases and excellent in one case; Table 5 ).

All cases of the direct “Steindler effect” were accompanied by static abnormal postures: severe pronation and wrist flexion contracture ( Table 5 ), while all cases of the reverse “Steindler effect” were accompanied only by fist clenching ( Table 5 ) and did not produce pronation, but only minor elbow extension contracture.

## Discussion

While the well-timed reinnervation via different nerve transfers 3 4 21 of the target muscles that provide the function of the highest priority, 1 EF, has become a routine procedure, 22 23 all types of tendon/muscle transfers 5 6 aimed at achieving the same goal have become rather “salvage procedure” at later stages of injury, myelo-/radiculo/plexo-/neuropathy or in an event of failed reinnervation. 5 6

Indications for any specific type of “salvage procedure” at later stages depend on the global/segmental functional status of the injured extremity for two main reasons: (1) through a long time after the trauma, the patient has already reeducated and adopted the preserved functionality of the injured extremity for the needs of daily living; hence, any change in the already entangled mechanics with tendon/muscle transfer can potentially lead to complete disability of the extremity; (2) the amount of preserved functions (neurological-wise) of the injured extremity does not always comply with the indications for any specific nonnerve-related procedure.

Indications for SF have dramatically narrowed along the historical road of the development of the procedure. 12 13 14 15 16 Improved surgical technique 12 13 14 15 16 has made it possible to reduce the number of complications of both static 19 and dynamic 19 origin associated with the procedure. Finally, the minimum requirements, that is, minimal amount of preserved segmental functions of the upper extremity, were reflected in the indications for successful SF. 19 According to Al-Qattan's scale, 19 these indications included a stable shoulder, near-normal or normal hand grip strength, near-normal or normal wrist and elbow extension strength, as well as the presence of the “Steindler effect” 19 . Some authors have pointed out the need for minimally preserved function of the primary elbow flexors. 19

In this study, the neurological status of the injured extremity of only three patients was in full compliance with AQIC ( Table 6 ). Two of them had a good and one patient had an excellent result after SF ( Table 6 ). Another five patients had a huge variety of AQIC at the time of inclusion ( Table 6 ), except for A3 criteria with 100% compliance ( Table 6 ). The exclusion of any other criterion than A3 (exclusion of one or more criteria) was associated with a different extent with the functional outcomes of SF.

**Table 6:** Correspondence rate of all included patients with Al-Qattan and study inclusion criteria and associated functional outcomes

#	Al-Qattan inclusion criteria a					Inclusion criteria (% corr.)	Result b	Inclusion criteria (% corr.)	Study inclusion criteria	
	A1	A2	A3	A4	A5				S1	S2 = A3
1	+	+	+	+	+	100% (5 of 5)	Good	100% (2 of 2)	+	+
2	+		+			40% (2 of 5)	Fair	100% (2 of 2)	+	+
3			+			20% (1 of 5)	Fair	100% (2 of 2)	+	+
4	+	+	+	+	+	100% (5 of 5)	Good	100% (2 of 2)	+	+
5	+	+	+	+	+	100% (5 of 5)	Excellent	100% (2 of 2)	+	+
6			+	+		40% (2 of 5)	Poor	100% (2 of 2)	+	+
7	+	+	+		+	80% (4 of 5)	Excellent	100% (2 of 2)	+	+
8		+	+	+		60% (3 of 5)	Good	100% (2 of 2)	+	+

Abbreviations: A1, strong elbow extension; A2, strong wrist extension; A3, strong hand grip; A4, stable shoulder; A5, preoperative “Steindler” effect; S1, flexor carpi radialis, flexor carpi ulnaris, pronator teres muscles M4 or higher on the MRC scale; S2, biceps brachii and brachialis muscles M0 on MRC.

a According to Al-Qattan 19

b According to Al-Qattan 19

*AQIC A1* ( Table 6 ): poor preoperative strength of the triceps brachii muscle disabled its influence as a counterforce (three cases of M2, M3, and M3 on the MRC scale) to the action of the transferred flexor-pronator mass across the elbow joint (proximal action). Therefore, active stretching of the transferred flexor-pronator mass was minimized during the entire rehabilitation period and was reflected in the residual EF contracture. Functional results in these cases were fair (No. 3), poor (No. 6), and good (No. 8; Table 6 ).

*AQIC A2* ( Table 6 ): Poor preoperative strength of the primary wrist extensors disabled their influence as a counterforce (three cases of M3, M2, and M2 on the MRC scale) to the action of the transferred flexor-pronator mass across the radiocarpal joint (distal action), which predetermined its “active insufficiency” 17 . This “active insufficiency” of the transferred flexor-pronator mass enabled its action across both the elbow (proximal action) and radiocarpal (distal action) joints, minimizing the maximum REF. Functional results in these cases were fair (No. 2), fair (No. 3), and poor (No. 6; Table 6 ).

*AQIC A4* ( Table 6 ): Three patients had glenohumeral joint instability at the time of inclusion. Two of them complied with two AQIC, and one complied with one AQIC. Functional results in these cases were fair (No. 2), fair (No. 3), and poor (No. 6) ;( Table 6 ).

*AQIC A5* ( Table 6 ): Overall, in the four cases with a positive preoperative “Steindler effect”, recovery of EF was considered excellent in two cases and good in another two cases ( Table 6 ). Three of them complied with all five AQIC, another one complied with four of the five AQIC, except for shoulder stability ( Table 6 ).

When analyzing the outcomes of a subgroup of five patients who failed to comply with all of the AQIC, it became obvious that only two criteria determined the final results of the Steindler procedure. Although all included patients showed the pretransfer ability of the flexor-pronator mass to act against resistance (all included patients complied with *AQIC A3,* Table 6 ), the quantitative expression of the outcome of the procedure depended on two main parameters. They included a mixture of transfer-mediated REF and residual posttransfer EF contracture. Both parameters were directly affected by the pretransfer functional status and, accordingly, the posttransfer counteraction of the antagonistic muscles of the upper arm (elbow extensor, triceps brachii muscle— *AQIC A1* ) and forearm (wrist extensors, extensor carpi radialis brevis and longus— *AQIC A2* ) in relation to the action of the transferred flexor-pronator mass.

**Fig. 2:**
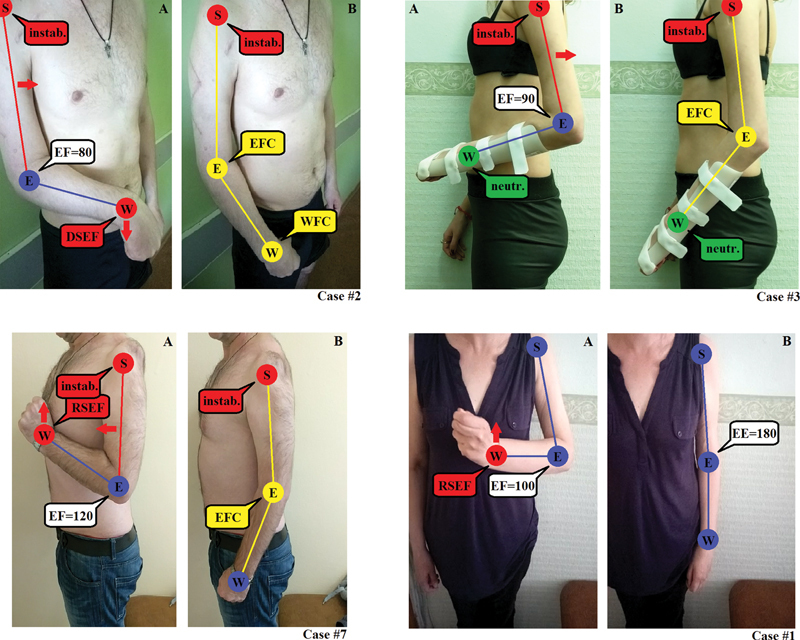
Clinical presentation of the patients after Steindler flexorplasty with different correspondence to Al-Qattan inclusion criteria, associated functional outcomes, static and dynamic (pathological locomotor phenomena – PLP) abnormal postures. Case#2: 40% correspondence to Al-Qattan inclusion criteria; Case#3: 20% correspondence to Al-Qattan inclusion criteria; Case#7: 80% correspondence to Al-Qattan Inclusion Criteria; Case#1: 100% correspondence to Al-Qattan Inclusion Criteria; ( **A** ) flexor-pronator mass flexes the elbow; ( **B** ) passive extension in the elbow joint; S, glenohumeral joint; E, elbow joint; W, radiocarpal joint; EF, elbow flexion; = number, range of motion in degrees; EE, elbow extension; EFC, elbow flexion contracture; WFC, wrist flexion contracture; instab., glenohumeral joint instability; neutr., neutral position; DSEF, direct “Steindler effect”; RSEF, reverse “Steindler effect”; red circle, dynamic instability of a joint or PLP; blue circle, dynamic stability, effective range of motion or absence of static abnormal postures; yellow circle, static abnormal postures; red arrow, direction of PLP.

The initially imperfect assessment of the functional efficacy of SF presumed the occurrence of residual EF contracture as the second major factor negatively affecting the final quantitative expression of the functional outcome of the procedure. In these cases, the “pure” REF, that is only the active component, regardless of the initial position in the elbow joint, mediated by the transferred flexor-pronator mass under its “active insufficiency”, on average barely reached 30 degrees (20 to 60 degrees; Table 5 ). Based on the results of this study, we believe that posttransfer EF contracture can be considered a positive factor. EF contracture in patients with no correspondence to *AQIC A1* ( Fig. 2 ) added another 10 to 50 degrees to the summarized REF (REF contracture or preflexion, plus “pure” REF), especially when flexor-pronator mass was acting under a condition known as “active insufficiency” due to partially dysfunctional wrist extensors (no correspondence to *AQIC A2* ). Accordingly, strong elbow extension should not be considered as a final inclusion criterion.

According to the results of this study, “active insufficiency” 17 of a transferred flexor-pronator mass can be overturned to “passive insufficiency” 17 by stabilizing the radiocarpal joint in a neutral position ( Fig. 2 : case No. 3) with an external orthotic device. This maneuver helped to produce another +10 degrees to the “pure” REF in case#3 (compared to case No. 2), which allowed the entire contractile potential of the transferred flexor-pronator mass to be transferred only across the elbow joint (proximal action) ( Fig. 2 ).The stabilization of the radiocarpal joint allowed partially neglecting noncorrespondence to *AQIC A2.*

Additionally, we believe that the instability of the glenohumeral joint ( *AQIC A4* ) had a minimal effect on the overall performance of the transferred flexor-pronator mass. In cases No. 2 and No. 3, forward flexion ( Fig. 2 ) and extension ( Fig. 2 ) in the glenohumeral joint, respectively, accompanied posttransfer EF due to partially preserved functions of either the anterior deltoid or the latissimus dorsi muscle. Overall, the fair performance of transferred flexor-ponator mass in both cases was due to the absence of both A1 and A2 AQIC ( Table 2 ). On the contrary, in an event of complete instability of the glenohumeral joint in case No. 7, in which there was full compliance with A1, A2, and A3 AQIC, the final outcome of SF was considered excellent ( Fig. 2 ). Accordingly, a stable shoulder should not be considered as a final inclusion criterion.

In this study, four adult patients were able to reproduce the preoperative “Steindler effect.” We believe that this was largely due to the preserved function of the brachioradialis muscle ( Table 2 ), a secondary elbow flexor whose isolated function was merely enough to flex the elbow but only assisted in holding the elbow flexed after gravity resistance had been overcome by a swinging maneuver. 19 We believe that the reproduction of the preoperative “Steindler effect” in adults with nonfunctioning brachioradialis muscle is rather impossible in comparison to the child population. This can be explained based on the simple lever physics: (1) the length of the effort arm 17 24 25 —the distance from the medial epicondyle to the elbow joint in children versus adults; (2) the length of the resistance arm 17 24 25 —the distance from the elbow to the radiocarpal joint in children versus adults; (3) load 17 —the weight of the entire forearm and hand with the elbow joint (fulcrum or pivot 17 ) lying in-between (first class lever 24 25 ) in children versus adults. As a result, a derivative of the increased length of the effort arm, the decreased length of the resistance arm load in children reduces the effort 24 25 produced by the tension of the flexor-pronator mass to balance the load torque, 24 25 allowing the child to reproduce the preoperative “Steindler effect” 19 . Accordingly, the presence of the preoperative “Steindler effect” can be neglected when considering SF in adults.

Most of the key activities of daily living (ADLs) require bimanual participation, 26 the roles of the dominant and nondominant upper extremity for these ADLs 26 are very different. For instance, children with cerebral palsy utilize a paretic upper extremity to assist in bimanual ADLs, wherein an unaffected upper extremity is used for unimanual ADLs. 26 It has been defined that the segmental functions of the intact upper extremity are more suitable for performing precision tasks (require fine motor skills 27 ), while the segmental functions of the paretic upper extremity are more suitable for performing assisting or complementary tasks (require less fine motor skills 27 ): holding and stabilising, 28 providing fixation, 27 etc.

Gates et al 29 analyzed the elbow-forearm angles (EF) required for the completion of unimanual key ADLs. It was defined that all tasks could be completed within REF = 121 degrees and all tasks required a minimum of REF = 81 degrees. 29 Seven (87.5%) out of eight patients in this study achieved REF = 80 degrees or greater ( Table 5 ), which was enough to provide effective assisting or complementary functions to the injured extremity. The only criterion that unified neurological characteristics to achieve an effective REF for assisting an injured upper extremity was sufficient function (M4 on the MRC scale) of the muscles of the flexor-pronator mass—study inclusion criterion S1 ( Table 6 ), 100% compliance among patients at the time of inclusion in the study.

All types of dynamic PLP in patients with good and excellent outcomes of the procedure were mainly associated with the necessity to actively overturn the “active insufficiency” of the transferred flexor-pronator mass to its “passive insufficiency” when flexing the elbow using primary wrist extensors and/or finger flexors. Fist clench, in contrast to the postoperative reverse “Steindler effect”, caused severe difficulties in utilizing all kinematic chains in the hand and wrist unit during uni- or bimanual ADLs in 100% of patients with good and excellent outcomes ( Table 5 , Fig. 2 : Case No. 7, Case No. 1). Opening and closing of the hand was almost impossible when reaching the maximum REF. Based on the results of this study, we state that the occurrence of PLP impairs the functionality of the hand and wrist unit and is an inevitable outcome in all cases of SF.

*Study limitations:* (1) a small number of included patients made it impossible to conduct any meaningful statistical analysis; (2) a huge variation in pathology and neurological status at the time of inclusion made it impossible to unify the indications for the procedure; (3) a huge variation in terms from the moment of dysfunction to inclusion in the study did not allow determining the influence of the time/outcomes factor; (4) a small number of patients with any type of primary or SNS did not allow determining the influence of partially recovered or lost (due to the harvesting of the donor nerve and the associated weakening of the corresponding muscles) functions on the final functional outcome; (5) since the change in the power of the transverse volar grip was not documented in this series, the characterization of dysfunctions of the kinematic chains in the hand and wrist unit associated with the transfer of the flexor-pronator mass was rather formal or descriptive.

## Conclusions

SF allows reliably restoring the unimanual upper extremity functionality in terms of effective EF among carefully selected patients. According to the results of this study, the functional success of the procedure primarily relies on two selection criteria (study inclusion criteria—SIC): (1) normal or near-normal function (M4 or higher on the MRC scale) of the muscles of flexor-pronator mass and (2) normal or near-normal function (M4 or higher on the MRC scale) of the primary wrist extensors. Therefore, a sufficient number of inclusion criteria required for unimanual functional success of SF can be reduced from five (according to the Al-Qattan's scale) to two (SIC). The last two should be considered obligatory.

SF may be indicated as a “salvage” procedure for patients who do not fully comply with SIC. Weakening or the absence (M3 or lower on the MRC scale) of the primary wrist extensors should not be considered a contraindication for the procedure. According to the results of this study, the restored REF in those patients helped to improve the assisting function of the affected upper extremity during bimanual activities of daily living. Therefore, when considering SF as a “salvage” procedure, a sufficient number of inclusion criteria can be reduced from five (according to the Al-Qattan's scale) to one (SIC).

The occurrence of dynamic PLP in the hand and wrist unit associated with the procedure is inevitable, since it has a direct negative effect on the opening and closing of the hand when the elbow is flexed. We believe that this procedure should be contraindicated in patients whose unimanual and/or bimanual activities of daily living require high functionality of the hand.
